# An intrinsic value system for developing multiple invariant representations with incremental slowness learning

**DOI:** 10.3389/fnbot.2013.00009

**Published:** 2013-05-30

**Authors:** Matthew Luciw, Varun Kompella, Sohrob Kazerounian, Juergen Schmidhuber

**Affiliations:** IDSIA/SUPSI/USILugano-Manno, Switzerland

**Keywords:** slow feature analysis, intrinsic motivation systems, norepinephrine, neuromodulation, exploration-exploitation

## Abstract

Curiosity Driven Modular Incremental Slow Feature Analysis (CD-MISFA;) is a recently introduced model of intrinsically-motivated invariance learning. Artificial curiosity enables the orderly formation of multiple stable sensory representations to simplify the agent's complex sensory input. We discuss computational properties of the CD-MISFA model itself as well as neurophysiological analogs fulfilling similar functional roles. CD-MISFA combines 1. unsupervised representation learning through the slowness principle, 2. generation of an intrinsic reward signal through learning progress of the developing features, and 3. balancing of exploration and exploitation to maximize learning progress and quickly learn multiple feature sets for perceptual simplification. Experimental results on synthetic observations and on the iCub robot show that the intrinsic value system is essential for representation learning. Representations are typically explored and learned in order from least to most costly, as predicted by the theory of curiosity.

## 1. Introduction

We describe a model called CURIOUSity-DRiven, Modular, Incremental Slow Feature Analysis (Curious Dr. MISFA), which autonomously explores various action contexts, learning low-dimensional encodings from the high-dimensional sensory inputs (i.e., video) that result from each such context. Autonomous behavior in this regard requires the coordinated interaction between a number of subsystems which enable an agent to balance exploration-exploitation, to engage in useful contexts while disengaging from others, and to organize representations such that newly learned representations do not overwrite previously learned ones. Ultimately, an agent making use of Curiosity Driven Modular Incremental Slow Feature Analysis (CD-MISFA) learns to seek out and engage contexts wherein it expects to make the quickest progress, learns an appropriate compact, context-dependent representation, and upon fully learning such a representation, disengages from that context to enable further exploration of the environment, and learning of subsequent representations. The goal of such an agent is to maximize intrinsic reward accumulation, and as a byproduct learn all such representations that are learnable given the contexts available to it. We not only show why the interacting subsystems of CD-MISFA are necessary for the kind of unsupervised learning it undertakes, but moreover, we show how the subsystems that enable the model to autonomously explore and acquire new sensory representations, mirror the functional roles of some of the underlying cortical and neuromodulatory systems responsible for unsupervised learning, intrinsic motivation, task engagement, and task switching.

Although difficult, attempts to integrate such disparate functional subsystems are not only helpful in understanding the brain, but are increasingly necessary for building autonomous artificial and robotic systems. It does not suffice, for example, to know the cortical mechanisms responsible for unsupervised learning of sensory representations, if these mechanisms aren't linked to the systems responsible for exploring one's environment. In the absence of external rewards, how should an agent decide which actions and contexts to explore, in order to determine which representations are relevant and learnable? If a sensory representation is deemed overly complex or even unlearnable, what are the mechanisms by which the agent can disengage from exploring its current context, in order to allow it to explore others? Although CD-MISFA is an algorithmic approach to developmental robotics, and does not explicitly model the neural mechanisms by which these functions are realized in the brain, it is notable that the functional roles of the various subsystems in CD-MISFA find counterparts in neurophysiology.

In the following, we first discuss background on CD-MISFA, Artificial Curiosity, and developmental learning, then provide a detailed computational description of how the various sub-systems in CD-MISFA operate and interact, followed by a description of the neurophysiological correlates whose functional roles mirror those of CD-MISFA; namely, the interactions between the neuromodulatory systems involved in intrinsic motivation, task engagement, task switching, and value approximation. CD-MISFA is implemented in two situations: an environment composed of synthetic high-dimensional visual contexts, and a real-world environment, with an actively exploring humanoid iCub robot. A method for measuring the learning cost in the different contexts is introduced, and it is shown that the model is most likely to engage within the context where it can learn an as yet unlearned representation, where the cost is least among all possible contexts; this type of behavior is predicted by the theory of curiosity, and may be a general principle of development. The second result shows that IM-based exploration enables the embodied agent to learn interesting sensory representations, again in the predicted order, all while operating on high-dimensional video streams as sensory input.

## 2. Curious Dr. MISFA

### 2.1. Background

#### 2.1.1. Artificial curiosity

Consider a setting in which an agent operates without a teacher or any other type of external motivation, such as external reward. In this case, an agent needs to be self-motivated, or *curious*. The *Formal Theory of Fun and Creativity* (Schmidhuber, 2006, 1991, 2010) mathematically formalizes driving forces behind curious and creative behaviors. This theory requires that a curious agent have two learning components: an adaptive predictor/compressor of the agent's growing history of perceptions and actions, and a reinforcement learner (Sutton and Barto, 1998). The learning progress or expected improvement of the compressor becomes an intrinsic reward for the reinforcement learner. To maximize intrinsic reward accumulation, the reinforcement learner is motivated to create new experiences such that the compressor makes quick progress.

#### 2.1.2. Curiosity and development

Such a creative agent produces a sequence of self-generated tasks and their solutions, each task still unsolvable before learning, yet becoming solvable after learning. Further, there is an expected order in task-learning. Since the value function of the intrinsic reward contains the cost of learning, in the sense of an estimation of what type of progress it can expect, a task with the lowest cost of learning is preferentially learned next, among all possible tasks.

An orderly acquisition of competence can be seen as a developmental process. An important aspect to development is the gradual emergence of more and more types of skills, knowledge, etc (Schmidhuber, 1997, 2002; Prince et al., 2005). Such emergence, referred to as developmental stages, can observed through behavioral competence (Lee et al., 2007). More specifically, by developmental stages we mean that certain competencies are always seen to precede later ones, although the earlier competencies are not necessarily prerequisites for those learned later (which would be the case in continual learning Ring, 1994).

It has been shown in an *n*-armed bandit scenario that a system based on Artificial Curiosity undergoes developmental stages (Ngo et al., 2011). Further, when the goal is to maximize expected improvement of the predictor or other world model, it was shown that it is *optimal* to concentrate on the current easiest to learn task that has not yet been learned Lopes and Oudeyer (2012) (also in a bandit scenario).

However, the bandit setting involves initial knowledge of the number of possible tasks, in which case the learner can initially reserve learning resources for each task. This is unrealistic for open-ended autonomous development, in which the number of different tasks is initially unknown. What is learned in one part of the environment could apply to another part of the environment. To enable open-ended learning, CD-MISFA learns one module at a time, and if it finds a context that is represented well by one of its already stored modules, it will not need to assign learning resources or time to that context.

#### 2.1.3. Developmental robotics

Developmental Robotics aims to discover and implement mechanisms that can lead to emergence of mind in a embodied agent (Lungarella et al., 2003). The underlying *developmental program* has several general requirements:

- *Not Task Specific.* The task(s) that the robot will handle, i.e., the skills that it can learn, are not explicitly coded in the program. In CD-MISFA, we have such a situation, as the perceptual representations that emerge are dependent on the statistics of the image sequences that are generated from autonomous exploration of the different contexts.
- *Environmental Openness.* Can the system handle a wide variety of possibly uncontrolled environments that the designers might not have explicitly thought of? Currently, CD-MISFA specifically requires a designer to define the environment contexts that the robot can explore over, and so this is a drawback of the system.
- *Raw Information Processing.* Learning is on raw (low-level) information, such as pixels and motor activation values. CD-MISFA slow features are updated directly from pixels, not symbolic inputs or hand-designed feature outputs. On the motor end, the active joints are extremely constrained, but this aspect is low-level as well.
- *Online Learning.* Batch data collection is avoided completely through the *incremental slow feature analysis (IncSFA)* technique, IncSFA (Kompella et al., 2012b), with which a perceptual representation is updated after each image.
- *Continual Learning Ring (1994)*. For scaling up the machine's intelligent capabilities, it is necessary that learned skills lead to (or are combined to create) more complex skills. CD-MISFA has not yet demonstrated this, but skill development (albeit in a limited sense) has been shown (Kompella et al., 2012a) to be enabled by its representation learning (i.e., exploiting the learned representations for external reward). Potentially, a framework for continual learning can be built in here; this is to be explored in future work.

With respect to development, a main contribution of this paper is to show how non-task-specific and low-level visuomotor interactions can give rise to emergent behaviors, which are at a higher (but not yet conceptual) level. In a set-up environmental context, an agent's randomly moving effectors (motor babbling) lead to observable consequences involving interactions with the environment not directly controllable by the agent. Slowness learning leads to the emergent “higher-level” representations, since the learning is forced to pay attention to the events that occur on the slower time-scale instead of the regular, but more quickly changing parts of the sensorimotor data stream. For example, a robot that watches its arm can learn causality between its joint controls and the images quite quickly, since there is an abundance of data in babbling — in a sense, this is highly salient. But for the robot to learn about something external to it (i.e., an object), that it interacts with more infrequently, without human supervision, is more difficult, but nonetheless enabled by slowness learning.

#### 2.1.4. Unsupervised visuomotor representation learning

There are many works on representation learning, but we are specifically interested in representation learning from high-dimensional image sequences where the sequence results from an agent's actions. Slow Feature Analysis (SFA; Wiskott and Sejnowski, 2002), is well-suited to this case. SFA applies to image sequences, and it provides *invariant* representations, unlike e.g., Principal Components Analysis (PCA; Jolliffe, 2005), which provides a compressed representation, but not invariance. SFA is also an appearance-based approach (Turk and Pentland, 1991; Murase and Nayar, 1995). Appearance-based approaches learn analog *world properties* (object identity, person identity, pose estimation, etc.) from a set of views. In the setting of a developmental embodied agent, SFA provides emergent invariant representations that resemble symbolic world knowledge; IncSFA provides this autonomously. When an agent is placed in the loop, such that its input sequence is caused by its selected actions, the emergent slow features have been shown to be useful decompositions of the environment (Mahadevan and Maggioni, 2007; Sprekeler, 2011), specifically for reinforcement learning (Sutton and Barto, 1998).

#### 2.1.5. Related works

Related to CD-MISFA in terms of having similar motivations and being based in developmental principles (Weng et al., 2001) are the biologically-constrained intrinsic motivation model, and robotic implementation, of Baldassarre et al. (2012)^1^, and the Qualitative Learner of Action and Perception (QLAP; Mugan and Kuipers, 2012). Powerplay (Schmidhuber, 2011; Srivastava et al., 2013) was also important in terms of motivating CD-MISFA.

The QLAP is a developmental robotics system designed to learn simplified predictable knowledge (potentially useful for skills) from autonomous and curiosity-driven exploration. It discretizes low-level sensorimotor experience through defining landmarks in the variables and observing contingencies between landmarks. It builds predictive models on the low-level experience, which it can use to generate plans of action later. It either selects its actions randomly or such that it expects to make fast progress in the performance of the predictive models (a form of artificial curiosity). A major difference between this system and ours is that we operate upon the raw pixels directly, rather than assuming the existence of a low-level sensory model. In QLAP, for example, the sensory channels are preprocessed so that the input variables track the positions of the objects in the scene. Through IncSFA, features emerge for raw visual processing, and this feature development is tightly coupled with the curiosity-driven learning.

The recently formulated PowerPlay can be viewed as a greedy variant of the Formal Theory of Creativity. In PowerPlay, an increasingly general problem solver is improved by searching for the easiest to solve, still not yet known, task, while ensuring all previously solved tasks remain solved. By its formulation, PowerPlay has no problems with *forgetting*, which can easily occur in an open-ended learning setup (Schaal and Atkeson, 1998; Pape et al., 2011). In CD-MISFA, when each new representation is learned well enough to be internally predictable (low error), it is frozen and added to a long-term memory storage, and therefore there will be no destruction of already learned representations. Further, CD-MISFA searches for the context corresponding to the easiest to encode new representation, thereby acting in a PowerPlay-esque manner.

### 2.2. CD-MISFA overview

CD-MISFA (Kompella et al., 2012a) combines *representation learning* with *curiosity-driven exploration*.

The agent autonomously explores among *m* contexts, and builds a *representation library*, denoted as

(1) $$\Phi^{ℒ}=\{\Phi_{1}^{ℒ},\Phi_{2}^{ℒ},\ldots ,\Phi_{n}^{ℒ}\}.$$

There are, lets say *n* (≤ *m*) different representations to learn in the environment (but the agent does not know *n*). So, one representation can suit more than one context. Learning resources are not assigned to each context individually. Instead, the agent learns one representation at a time.

Each representation $\begin{array}{c} \Phi_{i}{}^{ℒ} \end{array}$ is composed of two subsequent mappings. The first takes a sensory input vector (e.g., pixels) **x**(*t*) (where *t* indicates discrete time), and encodes it via slow features. A straightforward example is *linear* SFA, which projects **x** from *I* to *J* dimensions (*J* << *I*) via matrix **W** = (**w**_1_, …, **w**_*J*_), composed of *J* column vectors which are the slow features. In this case, for the *i*-th representation,

(2) $$\begin{array}{l} y^{i}(t)=x^{T}(t)W^{i}. \end{array}$$

The second mapping produces internal state *s*^*i*^ from slow feature output **y**^*i*^. To this end, it has a set of cluster centers **C** = (**c**_1_, …, **c**_ξ_) in the slow feature output space, and assigns the current state as the one with the smallest error from the current output:

(3) $$s^{i}(t)=\;\underset{j}{\text{arg min}}\|y^{i}(t)-c_{j}^{i}\|.$$

These mappings provide a simplification of the raw sensory data expected to be perceived when the agent is within the context. The first provides invariance, suppressing irrelevant information. The second provides specificity in the remaining (relevant) information.

#### 2.2.1. Contexts

**Figure 1:**
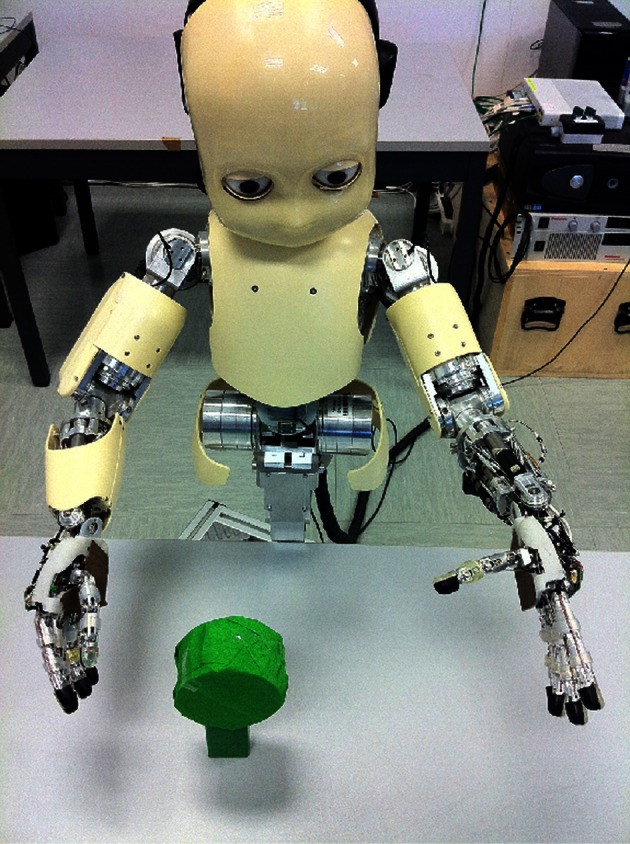
**Setup of an environmental context, in which the robot randomly moves its right arm (via single joint babbling).** The robot is not equipped with an object detection module, so it does not initially “know” about the object in its field of view. In each episode, the arm reliably displaces the object, and through training on this image data, a slow feature representation emerges that provides information about the state of the object (perturbed or not), invariant to what the robot already “knows” about (its shoulder joint settings).

The agent explores different *contexts*, by switching between them. Example contexts are rooms to explore, objects to interact with, or types of videos to perceive. As a specific example context, see Figure 1. We do not specifically define context, but note the following. (1) For convenience, a context can be thought of as having some state and action space, that is known to the agent. Thus, each context involves a set of states, a set of actions, and transition probabilities, from one state to another, given some action. (2) There is some *exploration policy*, internal to the context, by which the agent interacts with this environment. Exploration policies define how the randomized exploration (i.e., motor babbling) will occur on the given states and actions, e.g., Brownian motion on a mobile robot's wheel velocities with an innate reflex to turn away from obstacles sensed through the distance sensors (Franzius et al., 2007). (3) There is a potentially unobservable world state that defines the high-dimensional observations that will be the input to IncSFA. In Figure 1 is defined an example of a robot perched over an object. Here, a state space is a discretization of the right arm shoulder joint angles to 20 states, while the actions are (1) increase or (2) decrease the joint angle enough to move to an adjacent state. The world state includes the condition of the object, which is not known to the agent initially. But this becomes “known” through the slow feature encoding, after it is learned. Another example context is a simple grid world (Sutton and Barto, 1998) where the agent explores via random selection of one of four actions (up, down, left, and right). Its state space is given by the grid with observations of high-dimensional images showing the grid and the agent as viewed from above (Lange and Riedmiller, 2010; Luciw and Schmidhuber, 2012).

Each interaction with a context is an episode. There is a start condition to the episode, and an ending condition, which must occur at some point in the random exploration. After the ending condition, the agent must decide whether to continue exploration of this context, or to move to another^2^.

The agent uses curiosity to explore among multiple contexts. Rewards and motivation are intrinsic to the agent, and this intrinsic reward is calculated from representation learning progress. The agent can choose to remain engaged in its current context (exploitation), or seek to engage in another context (exploration). These decisions are due to utility judgements, where the utility is an estimate of *expected* learning progress of remaining engaged in the current context versus the expected learning progress of another contexts. If the former is higher, remaining engaged within the current context is most valuable, and, if the latter is higher, disengagement and switching is the more valuable choice.

**Figure 2:**
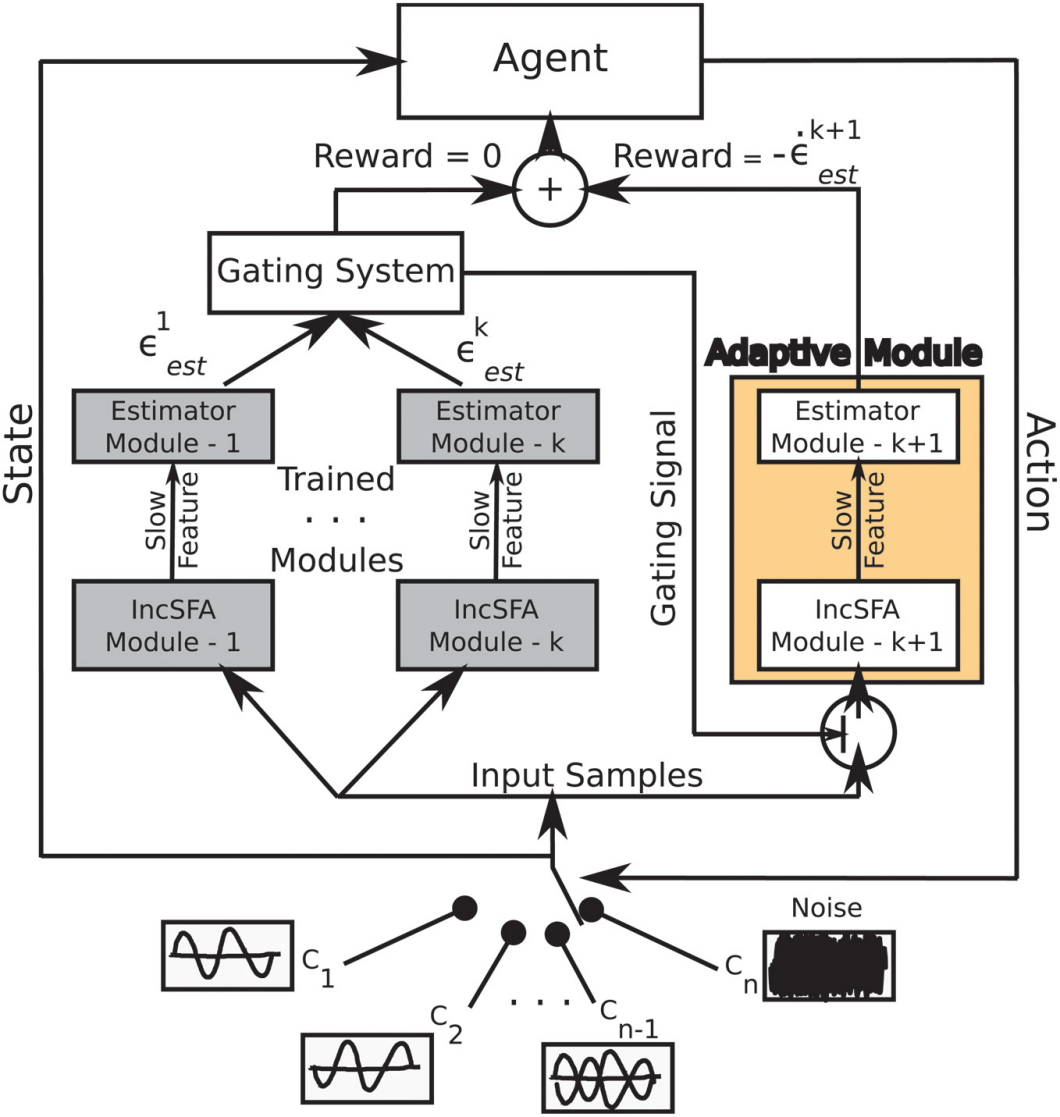
**Architecture of CD-MISFA**.

Figure 2 shows the architecture of CD-MISFA. The “adaptive module” encompasses the unsupervised learning part, which involves a combination of IncSFA and Robust Online Clustering (ROC). The representation library is shown by the “trained modules.” Estimation errors are denoted by ϵ, while intrinsic reward is denoted by $\dot{\epsilon }$. The intrinsic reward signal feeds into the value function estimation module. The possible environmental contexts are shown at the bottom, the current context is the “state” (with respect to the higher-level value function), while the “action” is either to remain engaged in that context, or to disengage and go to another.

Next, in Section 2.3, we discuss learning of a single representation. Specifically, we use IncSFA and the ROC method, respectively. Some details of these algorithms are described below, but more thorough descriptions can be found elsewhere (Guedalia et al., 1999; Weng et al., 2003; Peng and Yi, 2006; Zhang et al., 2005; Kompella et al., 2012b).

### 2.3. Unsupervised representation learning: IncSFA

SFA is concerned with the following optimization problem:

Given an *I*-dimensional input signal **x**(*t*) = [*x*_1_(*t*), …, *x*_*I*_(*t*)]^*T*^, find a set of *J* instantaneous real-valued functions **g**(*x*) = [*g*_1_(**x**), …, *g*_*J*_(**x**)]^*T*^, which together generate a *J*-dimensional output signal **y**(*t*) = [*y*_1_(*t*), …, *y*_*J*_(*t*)]^*T*^ with *y*_*j*_(*t*) := *g*_*j*_(**x**(*t*)), such that for each *j* ϵ {1, …, *J*}

(4) $$\Delta_{j}:=\Delta (y_{j}):=\langle {\dot{y}}_{j}^{2}\rangle \text{ is minimal}-$$

under the constraints

(5) $$\langle y_{j}\rangle =0\;(\text{zero mean}),$$

(6) $$\langle y_{j}^{2}\rangle =1\;(\text{unit variance}),$$

(7) $$\forall i<j:\langle y_{i}y_{j}\rangle =0\;(\text{decorrelation and order}),$$

with 〈·〉 and $\dot{y}$ indicating temporal averaging and the derivative of *y*, respectively.

The goal is to find instantaneous functions *g*_*j*_ generating different output signals that are as *slowly varying* as possible. The decorrelation constraint (7) ensures that different functions *g*_*j*_ do not code for the same features. The other constraints (5) and (6) avoid trivial constant output solutions.

In a linear sense, the optimization problem can be solved through an eigenvector approach, involving two uses of principal component analysis (PCA)—first, of the covariance matrix of the inputs (for whitening) and, second, of the covariance matrix of the whitened approximate *derivative* measurements (Wiskott and Sejnowski, 2002). IncSFA uses incremental algorithms for the two required PCAs. For the first, Candid Covariance-Free Incremental PCA (Zhang and Weng, 2001; Weng et al., 2003), is used, which can also reduce the dimensionality by only computing the *K* highest eigenvectors. For the second, Minor Components Analysis (MCA; Oja, 1992; Peng and Yi, 2006; Peng et al., 2007) updates the *J* slowest features.

The overall framework of IncSFA is shown in Algorithm 1. IncSFA needs to update the signal mean (learned incrementally by simple online average estimation), the *K* principal components, and the *J* slow features. In general *K* < *I* and *J* < *K* — and *K* and *J* are parameters of the algorithm. The learning methods use “amnesic” learning rate schedule, so they are potentially suited to non-stationary input sequences. IncSFA uses Hebbian (CCIPCA) and anti-Hebbian (CIMCA) update rules Dayan and Abbott (2001) to compute slow-features from a time-varying input signal.

**Algorithm 1:**
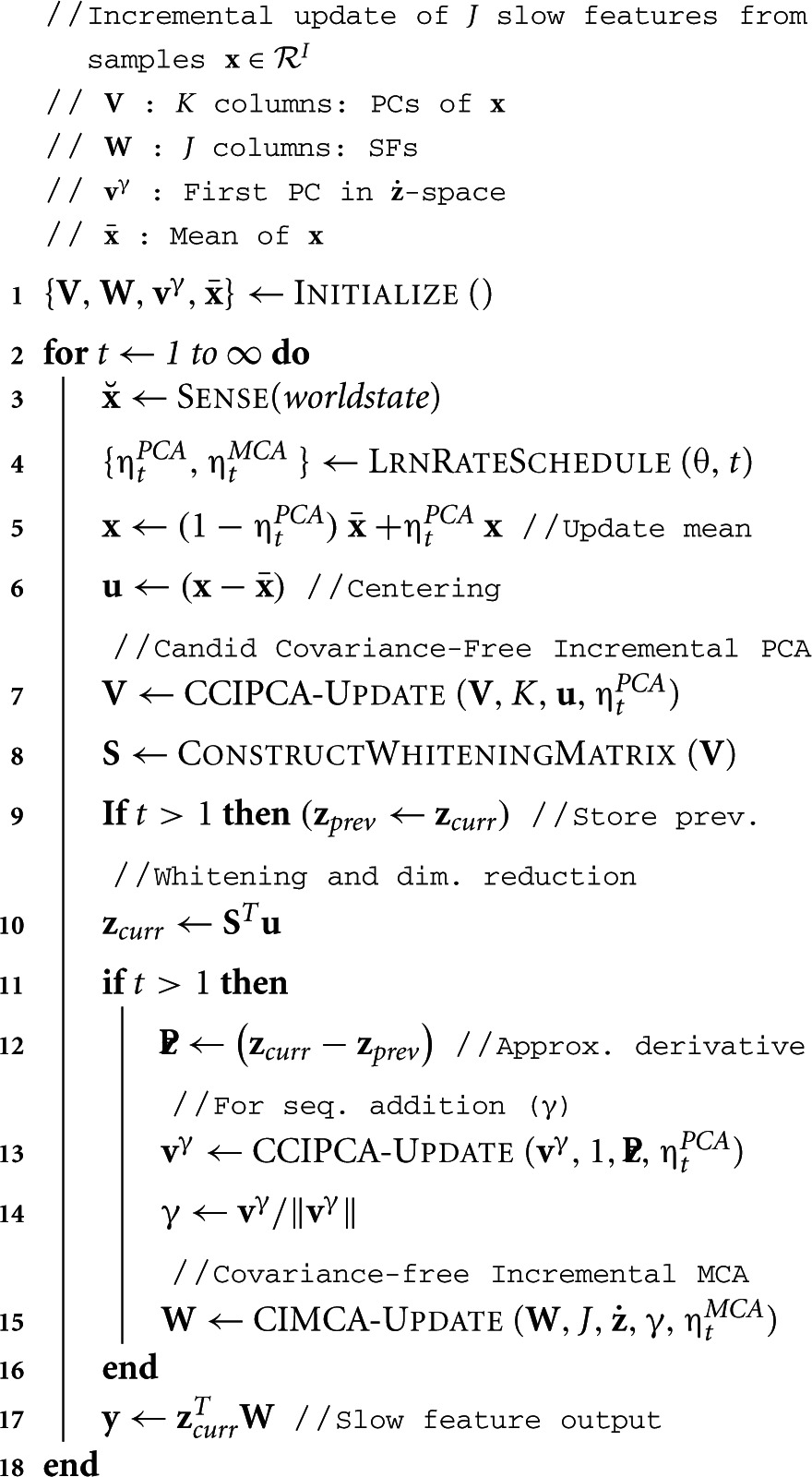
IncSFA (*J*, *K*, θ)

CCIPCA updates estimates of eigenvalues and eigenvectors from each centered observation. CCIPCA combines a statistically efficient Hebbian update with the residual method (Kreyszig, 1988; Sanger, 1989) to generate observations in a complementary space in order to update components besides the first, dealing with the requirement that any component must also be orthogonal to all higher-order components. The CCIPCA algorithm is presented in Algorithm 2. The principal component estimates are used to construct a whitening matrix. After whitening, the signal is (approximately) normalized and decorrelated.

**Algorithm 2:**
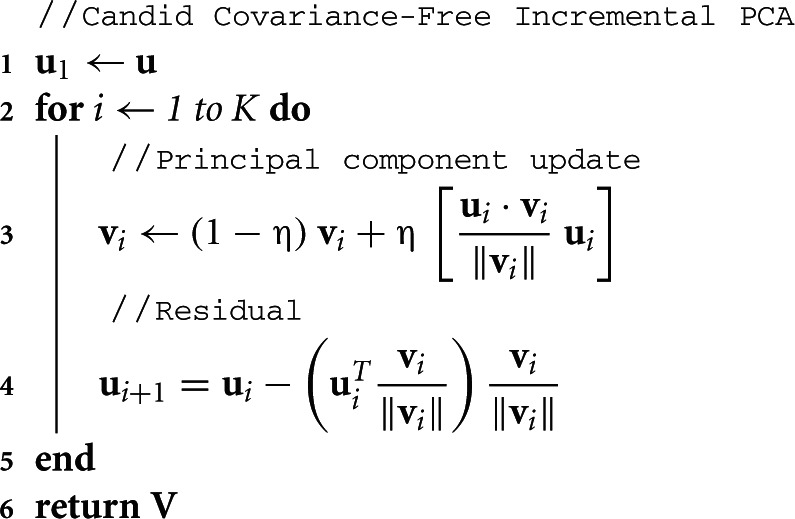
CCIPCA-Update (**V**, K, **u**, η)

Minor Components Analysis preferentially learns the least significant principal components. The update for each slow-feature vector **w**_*i*_ from 1 to *J*, is

(8) $$w_{i}\leftarrow (1-\eta^{MCA})w_{i}-\eta^{MCA}\left((\dot{z}\cdot w_{i})\dot{z}+\gamma \;\sum_{j}^{i-1}\left(w_{j}\cdot w_{i}\right)w_{j}\right)\;$$

where η_*MCA*_ is a learning rate. This update is based on anti-Hebbian learning with an additional Gram–Schmidt term inside the summation that enforces different features to be orthogonal. After updating, a feature is normalized for stability.

The feature output is an instantaneous function,

(9) $$y(t)=z{\left(t\right)}^{T}w(t).$$

### 2.4. Adapting the states with ROC

In a context's pre-defined state space, each state has its own instance of an online clustering algorithm. Clustering is done in an associative space that *combines* this pre-defined state space with the slow feature output space. These clusters, once learned, act as augmented internal states, potentially providing information about invariants captured with IncSFA.

As an example, consider again the robot viewing its arm move eventually toppling an object in the scene. The state space here is a quantization of the joint angles of the shoulder into 20 bins, thereby providing 20 states, leading to 20 instances of the clustering algorithm. A developed slow feature output here is a step function, e.g., when the object is not toppled, the feature output equals zero, and when the object is toppled the feature output equals one. Upon convergence of, first, IncSFA and, second, the clustering, each joint-angle state will be replaced by two internal states, which inform whether the object is or is not toppled.

Learning these clusters is not as straightforward as the above example makes it seem, since the signal is highly non-stationary during the early learning phases, due to its input being a function of adapting slow features. The slow feature outputs can change rapidly during the training phase. The estimator therefore has to be able to change its estimates to this non-stationary input, while converging to a good estimate when the input becomes stable. To this end, we use a clustering algorithm to specifically handle non-stationary data, called ROC (Guedalia et al., 1999; Zhang et al., 2005).

ROC is similar to an incremental K-means algorithm—a set of cluster centers is maintained, and with each new input, the most similar cluster center (the winner) is adapted to become more like the input. Unlike k-means, with each input, it follows the adaptation step by *merging* the two most similar cluster centers, and *creating a new cluster center* at the latest input. In this way, ROC can quickly adjust to non-stationary input distributions by directly adding a new cluster for the newest input sample, which may mark the beginning of a new input process.

But is this plasticity at the cost of stability? No. In order to enforce stability, clusters maintain a weight, which increases faster for more similar (to the cluster center) inputs. A large weight prevents a cluster center from changing that much. When two clusters are merged, their weights are also combined.

A sketch of the ROC per-sample update is in Algorithm 3. The ROC algorithm repeatedly iterates through the following steps. For every input sample, the algorithm finds the closest cluster *winner* and updates the center **c**_*winner*_ toward it, also increasing the weighting parameter *a*_*winner*_. Next, the closest two clusters are merged into one cluster. Then, a new cluster is created around sample **y**. Finally, all clusters weights decrease slightly. Parameters required are ξ, the maximum number of clusters, an amnesic parameter ϕ to prevent convergence, and the response function for similarity measurement.

**Algorithm 3:**
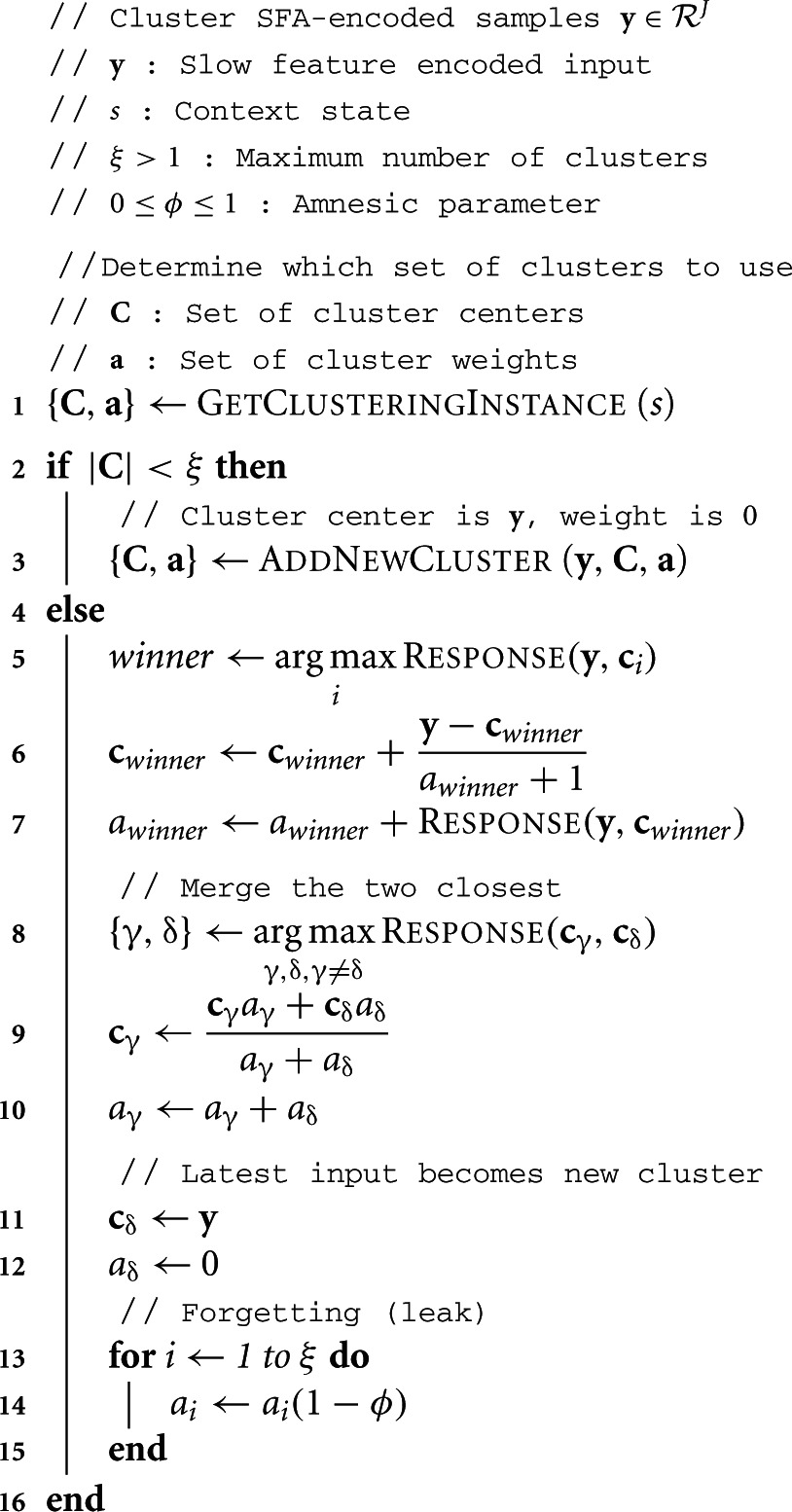
ROC-Amnesic(**y**, *s*, ξ, ϕ)

### 2.5. Intrinsic reward

**Figure 3:**
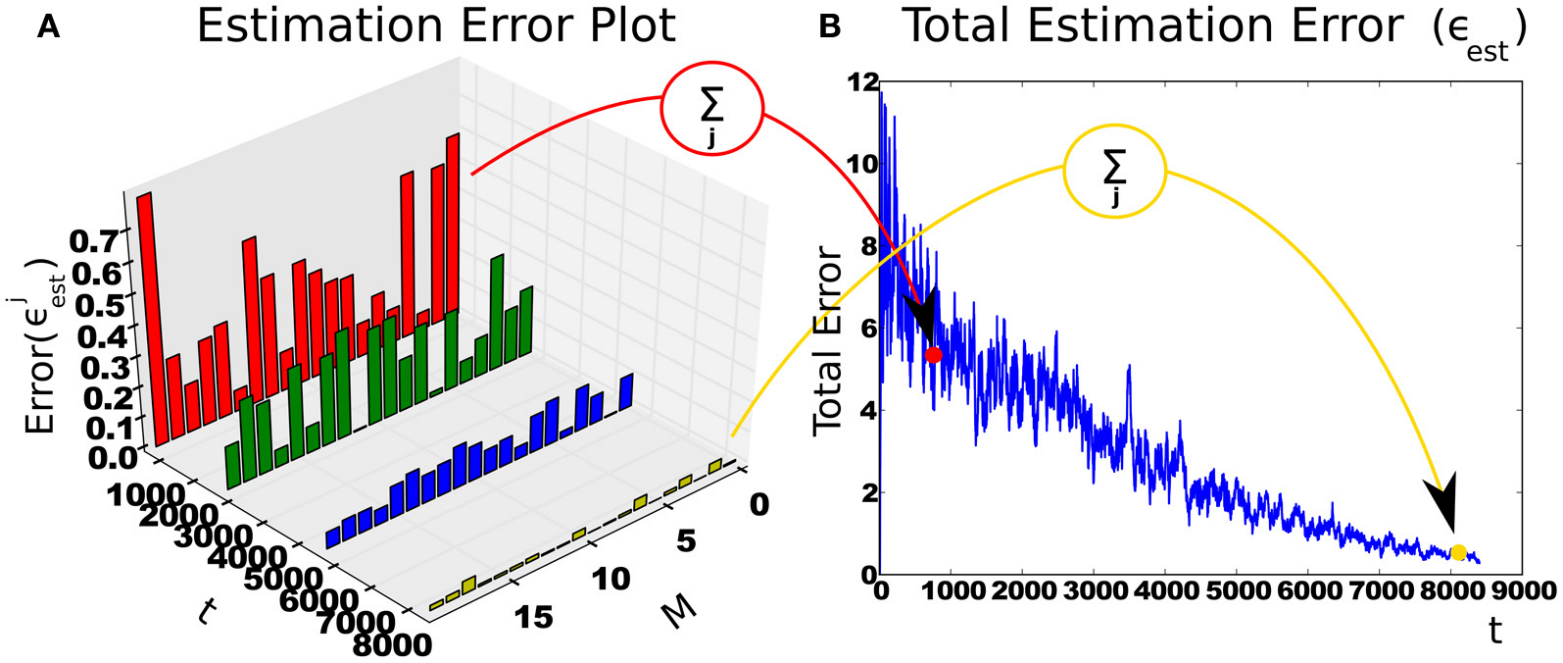
**Intrinsic reward is calculated from reduction of context-specific cumulative estimation error. (A)** The change in estimation error over time in a context with 20 states (M). With more experience, the features stabilize and estimator errors decrease. **(B)** The sum of estimation errors. The subsequent difference is the intrinsic reward.

The intrinsic reward is expected learning progress. Learning progress is approximated as the decrease in context-specific cumulative estimation error. Each context state *i* has an associated error ϵ^*i*^_*est*_. These errors are updated whenever the agent visits that state—

(10) $$\epsilon_{est}^{i}(t)=\underset{j}{\min}||y(t)-c_{j}||$$

where **y**(*t*) is the slow-feature output vector and **c**_*j*_ is the *j*-th cluster center associated with this state. The context's current estimation error is the sum of stored errors, over all *M* context states,

(11) $$\epsilon_{est}(t)=\sum_{i=1}^{M}\epsilon_{est}^{i}(t),$$

and the intrinsic reward is the *derivative* of the total estimation error ${\dot{\epsilon }}_{est}=\epsilon_{est}(t)-\epsilon_{est}(t-1)$. Figure 3 shows an example with a 20-state estimator.

### 2.6. Module storage and gating

Once the slow feature outputs stabilize, the estimator clusters converge and the error will become very low. Next, estimator clusters with small weights *a*_*i*_ are eliminated, to avoid having spurious internal states. Finally, this overall representation *module* is frozen, considered *learned*, and placed in long-term memory.

The already trained set of modules are the abstraction library $\begin{array}{c} \Phi^{ℒ} \end{array}$ (Equation 1). If one of these module's estimation error within a context is below a threshold, that context is assigned that module's representation and the adaptive training module will be prevented from learning, by this gating signal. There will no intrinsic reward in this case. On the other hand, if the estimation error of all the trained modules for the incoming data is above the threshold, the gating signal enables the single adaptive module to be trained on the input data. Hence the training module will encode only data from input streams that were not encoded earlier.

### 2.7. Engage/disengage mechanism

Every time the agent exits a context, the agent needs to make a decision. To this end, the agent can take two *internal-actions*, $A^{o}=\{\text{engage, disengage}\}.$. The internal-action *engage* allows the agent to stay in the same context (starting over), while *disengage* causes the agent to switch to another context. For the purposes of our model, we do not allow the agent to select the context it will switch to, instead having it randomly selected. Thus, the transition-probability model *P* of the internal environment (modeling transition probabilities between all pairs of contexts *i* and *j*, conditioned on the two internal-actions) is given by:

(12) $$P_{ij}^{engage}=\{\begin{array}{ll} 1, & \text{if }i=j\\ 0, & \text{if }i\neq j \end{array}$$

(13) $$P_{ij}^{disengage}=\{\begin{array}{ll} 0, & \text{if }i=j\\ \frac{1}{N-1}, & \text{if }i\neq j \end{array}$$

∀*i*, *j* ϵ [1, …, *N*].

### 2.8. Reward and value function

The agent maintains an estimated *reward function*, which is the expected change in estimation error when transitioning from context *o* to context *o*′ (and *o* = *o*′ is possible). The agent's reward function is updated at every engage-disengage decision, from the intrinsic rewards, as a sample average:

(14) $$R_{a}^{o,o^{\prime }}:=(1-\eta )\;R_{a}^{o,o^{\prime }}+\eta \sum_{t}^{t\;+T}-{\dot{\epsilon }}_{est}(t)$$

where 0 < η < 1 is a learning rate, *T* was the duration of the previous context until its termination, (*o*, *o*′) ϵ {*O*_1_, …, *O*_*n*_} and *a* ϵ {engage, disengage}.

Using the current updated model of the reward function *R* and the internal-state transition-probability model *P*, the agent's policy ($O\times A^{o}\to [0,1]$) is updated.

It is important that the policy adapts quickly enough to adapt to the quickly changing reward function. Intrinsic rewards can change quickly as learning progresses, and the RL must adapt quicker than the underlying representation learner. We used model-based Least Squares Policy Iteration (Lagoudakis and Parr, 2003), which is an efficient value-estimation technique, although in principle the more biologically-plausible temporal-difference (TD) methods could also work.

#### 2.8.1. Epsilon-greedy

The agent cannot take the value-maximizing decision from the very beginning, since it needs time to build its value estimates to a more accurate level. Early on, it can make decisions more or less randomly so that it can gather experience in the different contexts, and to learn good estimates of value over all contexts. Given good value estimates, it can choose to engage within the context where it should learn quickly, in other words, make the fastest learning progress, and to lead to a quick learning of the next representation. To this end, the model augments its internal action selection with decaying ϵ-greedy exploration.

## 3. Neural correlates to CD-MISFA

### 3.1. SFA and competitive learning—entorhinal cortex and hippocampus

Slow Feature Analysis variants have been used to simulate representation learning in a number of biological scenarios. Based on the general principle that underlying driving forces manifest through slow changes in sensory observations, the features that emerge from SFA often encode important *invariants*. Hierarchical SFA has been shown to develop *grid cells* from high-dimensional visual input streams (Franzius et al., 2007). Grid cells, found in entorhinal cortex (EC) (Hafting et al., 2005), have a pattern of firing that effectively represent hexagonal codes of any two-dimensional environment. As such, grid cells are effective *general* representations for spatial navigation in typical environments.

A competitive learning layer, over the top-layer of slow features, leads to features acting as *place cells* or *head-direction cells*, depending on what changes more slowly from the observation sequences. A place cell will fire when the animal is in a specific location in the environment, typically invariant to its heading direction. Head-direction cells fire when the animal faces a certain direction, no matter what coordinate position it is in. Place cells and head-direction cells are found in hippocampus (O'Keefe and Dostrovsky, 1971; Taube et al., 1990), which has input from EC. It's been hypothesized that hippocampus acts as a relatively fast encoder of specific, episodic information, on top of cortex, which learns general structure from lots of data over a long period (Cohen and O' Reilly, 1996)—“It has been proposed that this universal spatial representation might be recoded onto a context-specific code in hippocampal networks, and that this interplay might be crucial for successful storage of episodic memories (Fyhn et al., 2007).”

SFA's biological plausibility was furthered by IncSFA, which avoids batch processing and has Hebbian and anti-Hebbian updating equations. Hierarchical SFA (Franzius et al., 2007) and IncSFA (Luciw et al., 2012), with competitive learning on top, was shown to develop place and head-direction cell representations. For the representations learned in CD-MISFA, we use the basic structure suggested by these results: A slow feature learner (possibly hierarchical) for *global* features (IncSFA), inputs into a competitive learner for development of *local* features (ROC).

### 3.2. Neuromodulatory subsystems for intrinsic reward and context switching

#### 3.2.1. Intrinsic rewards: dopamine and learning progress

Dopaminergic projections originate from the ventral tegmental area (VTA). Dopamine has been implicated in reward prediction (Schultz et al., 1997), leading to plausible relation to the theory of reinforcement learning (Sutton and Barto, 1998)—specifically, dopamine may be acting as a TD error signal. However, this account remains controversial (Redgrave et al., 1999; Kakade and Dayan, 2002). A major deviation from the dopamine as TD-error theory comes from data implicating dopamine in responding to novel salient stimuli (Schultz, 1998; Redgrave and Gurney, 2006), even for stimuli that are not predictive of reward. Dopaminergic responses to such stimuli fade over subsequent trials. It has been proposed that this characteristic serves the purpose of a “novelty bonus”—e.g., a reward addendum serving as a “optimistic initialization.”

These data present intriguing correlations to the curiosity theory. Dopamine release in response to novel stimuli could potentially signal a predicted intrinsic reward—an expectation of *learning progress*. Could DA in some situations signal the intrinsic reward? Dopamine's potential role in intrinsic motivation has been discussed before (Redgrave and Gurney, 2006; Kaplan and Oudeyer, 2007), but not with respect to the formal theory of curiosity Schmidhuber (2010), which predicts that intrinsic reward should be proportional to compression progress. Computational models in neuroscience often treat intrinsic reward as resulting from the novelty of a stimulus. If intrinsic reward really does result from novelty, we would expect persistent high levels of dopamine in response to unpredictable noisy stimuli (as it remains novel from moment to moment). On the other hand, if intrinsic rewards encode compression progress, we would expect decreases in the level of dopamine as the predictive model becomes unable to learn anything more about the structure of the noise^3^.

#### 3.2.2. Engagement and disengagement (and switching): norepinephrine

Neurons of the locus coeruleus (LC), in the brainstem, are the sole source of norepinephrine (NE). NE is linked to arousal, uncertainty, vigilance, attention, motivation, and task-engagement. The LC-NE system is more traditionally thought to affect levels of arousal, but more recently has been implicated in optimization of behavioral performance (Usher et al., 1999; Aston-Jones and Cohen, 2005; Sara, 2009).

In that context, the activity of the LC-NE system can be understood as modulation of exploration-exploitation. The tonic differences in LC-NE response are associated with levels of arousal. Tonic NE response is correlated with task performance levels (Usher et al., 1999). Low tonic activity coincides with low attentiveness and alertness (Aston-Jones et al., 1991), while high tonic activity coincides with agitation and distractibility (Aston-Jones and Cohen, 2005). Good task performance coincides with an intermediate tonic level during which phasic bursts of activity are observed, while poor task performance due to distraction is associated with high tonic activity. In phasic mode during periods of intermediate tonic NE activity, NE is released in response to task-relevant events (Dayan and Yu, 2006). As suggested by Usher et al. and others (Usher et al., 1999; Aston-Jones and Cohen, 2005), the phasic modes might correspond to exploitation, whereas high tonic states of NE activity might correspond to exploration.

When it is beneficial for the agent to remain engaged in the current task, the tonic NE level stays moderate, and only relevant task stimuli will be salient. However, when it is not beneficial to remain engaged in the current task, the NE level raises and task-irrelevant stimuli become more salient. This drives the agent to distractibility, and task performance suffers. Attending to some distractor stimuli could have the effect of causing the agent to switch to another task in which this distractor becomes relevant, ostensibly with the purpose of exploring among available tasks (i.e., it “throws the ball in the air so another team can take it” Aston-Jones and Cohen, 2005).

In CD-MISFA, the agent's two internal-actions, (engage or disengage), and the reasons they are taken, links to the NE-driven task engagement/disengagement model. Boredom (low NE) indicates that a good representation already has been learned, leading to low estimation error, and thereby low potential intrinsic reward. Distractibility (high NE) indicates that the errors are too high, not decreasing quickly enough, or they cannot be reduced. In this case, it becomes valuable to disengage and find some other context, where learning may progress faster (or at all). When the agent has found a good context, the estimation errors decrease regularly, providing intrinsic reward that leads to a high value estimate (and a desire to remain engaged in that context).

### 3.3. Frontal cortex: value function and representation selection

The NE and DA neuromodulatory systems each have reciprocal connectivity with the prefrontal cortex—executive areas, which deal with cognitive aspects such as decision making, and top-down control of other functions, such as selective attention (Miller, 2000). If the LC-NE system is handling task-engagement and disengagement based on some value judgement, then this system needs to be controlled by another system that is estimating these values. The prefrontal cortex (PFC) plausibly plays a role in value estimation, and might use the utility information to provide top-down regulation of the activities of the LC neurons (Ishii et al., 2002).

PFC and nearby structures, specifically the anterior cingulate cortex (ACC) and the orbital frontal cortex (OFC), are implicated in value-based judgements. The ACC is involved in error detection (i.e., recognizing a prediction error) and estimating the costs of these errors (Bush et al., 2002). OFC is thought to be of import in motivational control of goal-directed behaviors (Rolls et al., 1996)—OFC damage leads to responses to objects which are no longer rewarding (Rolls et al., 1994; Meunier et al., 1997). The dorsolateral pre-frontal cortex (DLPF) is implicated in value-based working memory (Rao et al., 1997). Thus, these structures could possibly work together to estimate a value function, in the RL sense (Ishii et al., 2002).

Another important property of PFC is to maintain an appropriate task representation, i.e., imposing internal representations that guide subsequent performance, and switching these for another when it is no longer appropriate (Miller, 2000; Cohen et al., 2004). This property requires mechanisms to keep goal-relevant information (i.e., what should be considered salient and what should be considered a distractor) enabled in resonance with lower structures. Further, it requires a mechanism to maintain a context despite bottom-up disturbances, and a mechanism to switch the context. The PFC has connections from and to higher-order associative cortices, so it is in a good position to impose task-relevant representations from the top-down. Such “executive attention” enables memory representations to be “maintained in a highly accessible state in the presence of interference, and these representation may reflect action plans, goal states or task-relevant stimuli in the environment (Kane and Engle, 2002).”

## 4. Experiments and results

### 4.1. Synthetic signals

In other works, we have studied the types of representations uncovered by IncSFA, and their applicability (Kompella et al., 2012b; Luciw and Schmidhuber, 2012). The experiments here will focus moreso on the curiosity-driven behavior, especially in comparison to what the formal theory of curiosity predicts. We also explore the potential link of CD-MISFA to neuromodulatory task-switching—what quantities in our experimental results might be analogous to associated neuromodulators dopamine and norepinephrine?

CD-MISFA's typical behavior involves cycles of exploration, exploitation, and module storage. Exploration involves context switching, enabling accumulation of learning progress estimates about each context. The exploitation period has it settle into a single context where progress is easiest, until the representation is stored in long-term memory. Based on the formal theory of curiosity, we expect CD-MISFA to learn the representations in inverse order of their learning difficulty. Further, it will not waste time on anything unlearnable, corresponding to noise—which we note is novel and surprising in the traditional sense of Shannon et al. (1949), however, uninteresting since no learning progress can be made.

To this end, the first experiment involves a synthetic learning environment, with four types of *sources*—also known as *driving forces* Wiskott (2003). The simple driving forces are the fundamental “causes” of the complex observations. For example, an observation sequence given by an onboard camera of a mobile robot is “caused” by the robot's position, orientation, and camera angle. One cannot reconstruct the observations from the driving forces alone, of course, but tasks and rewarding conditions are often associated with the driving forces, and knowledge of the driving forces leads to useful (potentially rewarding) predictive power.

**Figure 4:**
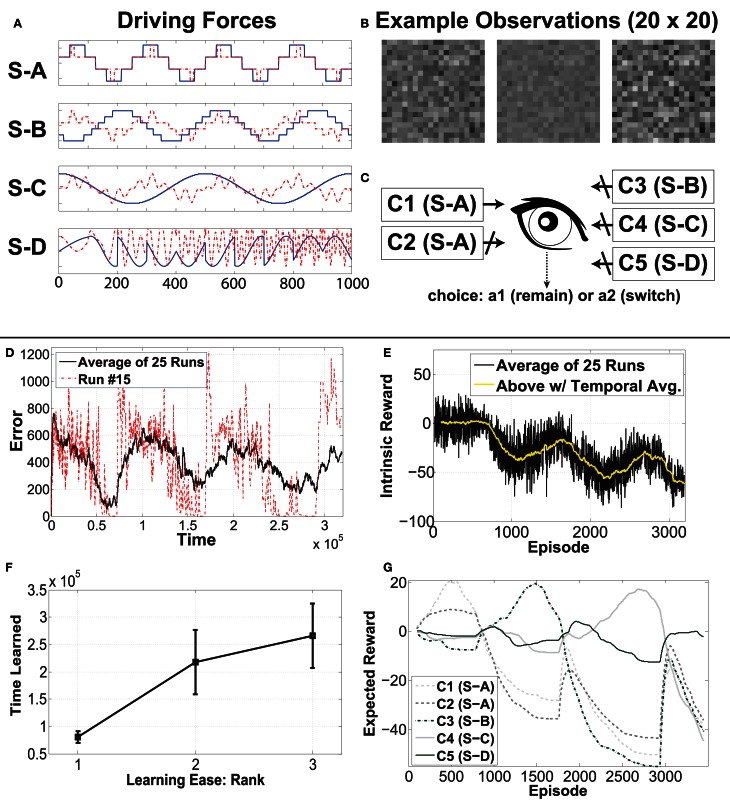
**Experimental setup and results of Curious Dr. MISFA with synthetic data.** See text for details.

At any time the agent is experiencing one of five contexts. Two contexts are generated based on the same driving force, while the other three each have a different driving force. In Figure 4A, the 2 × 1000 (dimension by time steps) signal sources can be seen (S-A, S-B, S-C, S-D), ordered via learning difficulty, with the easiest signal at the top. The blue curve shows the first dimension, while the red dotted curve shows the second dimension. At the bottom, we have a highly non-stationary source, which changes irregularly, so as to be unlearnable to IncSFA. We want to hide each of these sources within a different high-dimensional process, albeit linearly, so that linear IncSFA will be able to extract them and it will take enough effort to do so. A high-dimensional observation is generated from a source by multiplication with one of four 400 × 2 matrices, which are randomly generated before each experiment. The 400 resulting values are rearranged into a 20 × 20 and value-normalized from zero to one to be pixel values for each image. Each input observation **x**(*t*) is an image of 20 × 20 pixels. In Figure 4B, one can see a few sample observations. The task for CD-MISFA is to extract all three learnable driving force signals from a single stream of high-dimensional observations.

Figure 4C shows the CD-MISFA agent's environment, which contains the five contexts (C1–C5; which can be considered states in the RL sense), and has two actions—stay (engage) or switch (disengage). Each time a context is entered, 100 steps of observations are fed to IncSFA. Each context has a local clock, so that the local time step will pick up where it left off if the agent returns from another. At the end of the 1000 time steps, the local time step resets^4^.

#### 4.1.1. Measuring learning difficulty

In order to test predictions of the Formal Theory of Curiosity, we need to analytically establish a definition of learning cost for slow features, by which we will measure the relative complexity of the signals within each context. We introduce here a measure denoted as Ω, to quantify the learning progress of IncSFA.

(15) $$\Omega (x)=[1-\frac{\eta^{mca}(\lambda_{n-1}-\lambda_{n})}{1-\eta^{mca}-\eta^{mca}\lambda_{n}}]$$

where λ_*n*_, and λ_*n* − 1_ are the eigenvalues corresponding to the lowest-order and second lowest-order (respectively) principal components in the whitened derivative space. We will discuss the origins of Ω further in Section 4.4, with full derivation.

In this experiment, the three learnable signals are quantified as Ω^*A*^ = 0.9933 (for S-A), Ω^*B*^ = 0.9988 (for S-B), and Ω^*C*^ = 0.9997 (for S-C). They are quite close due to the similarities of the last and second to last eigenvalues in each distribution, however, there is a non-linear relationship between Ω and learning time. For S-A, about 2–3 epochs are needed. For S-B, about 15 epochs are needed. For S-C, about 40 epochs are needed.

#### 4.1.2. Experiment setup

The experiment setup is as follows. Since there are 1000 different time-steps, we use 1000 states for the clustering. Thus, there will be 1000 different clusterers, each with maximum number of clusters set as 2. The estimator error is measured as an average of the estimation errors after each episode—an interaction with a single context of 100 time steps. The intrinsic reward estimates and policy are updated after each episode. Once the estimation error gets below the threshold 0.3, the module is frozen, and a new module created. The initial setting for ϵ-greedy is 0.6, which decreases via multiplication with 0.995 after every episode, and is reset when a new module is created. The learning rates for IncSFA: for CCIPCA, a 1/*t* learning rate is used, with amnesic parameter *l* = 2 (Weng et al., 2003), the MCA learning rate is a constant η_*MCA*_ = 0.05. We collected results over 25 different runs. Each run has a different initializations of all aspects, wherein CD-MISFA operates for time 3.5 × 10^5^.

We note here some implementation details about the gating system. The gating system prevents corruption of the adapting IncSFA with samples from an already known/learned representation. This is implemented as a buffer that fills during each episode, at the end of which the 100 observations are sent to all feature sets, from which the output is calculated. That output is then sent to the clusters in each SF output space, enabling error calculation for all modules. If the minimum module error is less than 0.3, the previous 100 samples are not used for learning, and a negative reward of −100 given. Otherwise, the samples are fed to IncSFA for learning. In this case, the intrinsic reward is calculated as the difference between the current estimation error of the adaptive learner and the same context's previously measured estimation error. The negative reward serves only to speed up the learning process. If it were removed, each run would simply take longer to complete.

#### 4.1.3. Behavior

In all 25 runs behavior of CD-MISFA involved alternating phases of mostly exploration among all contexts, and exploitation once it settles on a context where it expects to make the most progress. We will call this exploitation-exploration process a cycle. Exploration is caused by the initial high amount of change in the adapting slow features, so that the estimator, which is on top of the slow features, cannot make progress. Once CD-MISFA remains within a context for enough time, the features become predictable enough so that an advantageous intrinsic reward can result. Due to ϵ-greedy, it continues to switch between contexts, allowing it to accumulate good estimates for all (the previous intrinsic reward accumulations are captured in the reward function). As ϵ decays, the policy converges to the simple but optimal strategy to disengage from all contexts except the easiest to learn new context.

#### 4.1.4. Results

Results are shown in Figures 4D–G.

Part **(D)** shows the average cumulative estimator error (a single run is also plotted for perspective). In each cycle, the error starts high, then trends down as representations are learned; finally a module is created. Within each run, this is a rather noisy signal, as the agent jumps from context to context. The end of each run has only the unlearnable context remaining, so the error cannot reduce enough to store another module.

Part **(E)** shows the run-averaged and temporally-averaged (for smoothness) intrinsic reward. Each cycle (notably except the first) involves a rise and fall. Relatively low intrinsic reward that trends higher is associated with disengagement behavior. Relatively high intrinsic reward that trends lower is associated with engagement behavior. The high punishment for boring experiences within a learned context tends to drag the values down, moreso later in each run, when more representations have been learned. The first cycle seems to lack the typical rise, which we posit is due to the simplicity of the signal.

Part **(F)** shows average learning times and standard deviations for the three learnable signals. The ordering tends to be as predicted, but not always: A module for S-A emerges first all 25 times, S-B's module occurs second 18 times, and third 7 times, while S-A's module is mostly third (18/25). Due to the 7 runs when S-B and S-C were learned opposite as expected, the average learning time for S-B is higher than the average time when the second module is typically learned (as can be seen in Figure 4D), and the average learning time for S-C is lower than when the third module is learned.

Part **(G)** illustrates the reward function for run number 15, which is a fairly typical run. C1 and C2 are associated with initial rising reward. Once the shared source (S-A) is learned, both have their expectations of reward drop. We see C3 subsequently rise, followed by C4, then C5 (unlearnable).

#### 4.1.5. On invariance

**Figure 5:**
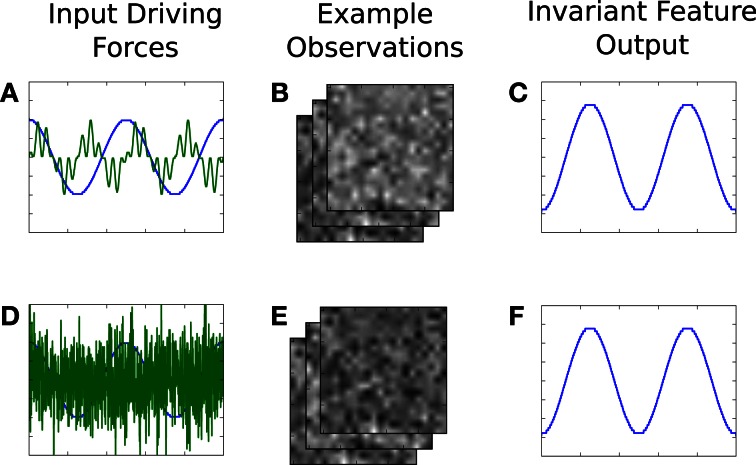
**An illustrative example of invariance, in the context of our synthetic signal experiment.** A two dimensional driving force **(A)** generates high-dimensional observations **(B)**, from which IncSFA learns features that extract the original driving force. **(C)** The output of the first, slowest, feature. After learning, the second part of the driving force is replaced by noise **(D)**, causing different images **(E)**. However, the previously learned first feature output does not change **(F)**.

There are two independent dimensions to each source, which together generate the observations. The corresponding representation thereby also contains two parts. One part of the driving force is (trivially) invariant to the other part, and, after learning, the invariance property is observable at the representation outputs. For example, if (after learning) the first dimension of our source is held constant but the second allowed to change, then the observations will change, but the output of the first feature of the corresponding component will be constant, while the second changes. Figure 5 illustrates this concept. As a real world example, consider place cells and head-direction cells. The output of the place cells are invariant to changes in orientation, and vice versa.

#### 4.1.6. On neuromodulators

The estimation error profile observed in Figure 4D and associated behavior mirrors the findings regarding the LC-NE system and the “inverted U.” High levels of estimation error correspond with predominantly disengagement and switching (“agitation”), while low levels of estimation error correspond with switching (“boredom”). There is a “sweet spot” of error, where the agent mostly engages in a single context. In this sweet spot, the intrinsic reward, representing learning progress, is at its relative peak. The intrinsic reward signal could link to dopamine, although, as we mentioned, there is no conclusive evidence about this.

### 4.2. Emergent representation from sensorimotor loops—an iCub experiment

This experiment uses an embodied agent (iCub) with real high-dimensional images (grayscale 75 × 100), from the robot's eyes. There are two contexts here. In each, the iCub explores via random movement of its shoulder joint, causing the outstretched hand to eventually displace the single object in its field of view. It then observes the outcome while the hand continues to move. It is not given any prior knowledge about the objects, itself, or any concepts at all. It merely observes the pixel values, and uses CD-MISFA for learning and decision making. In one context, the object is a cup, which topples over upon contact with very predictable outcome. In the other, the object will roll in different directions. About 70 episodes of image sequences were collected for each context. The eventual slow features, emergent from the holistic images, will code for the state of the objects.

**Figure 6:**
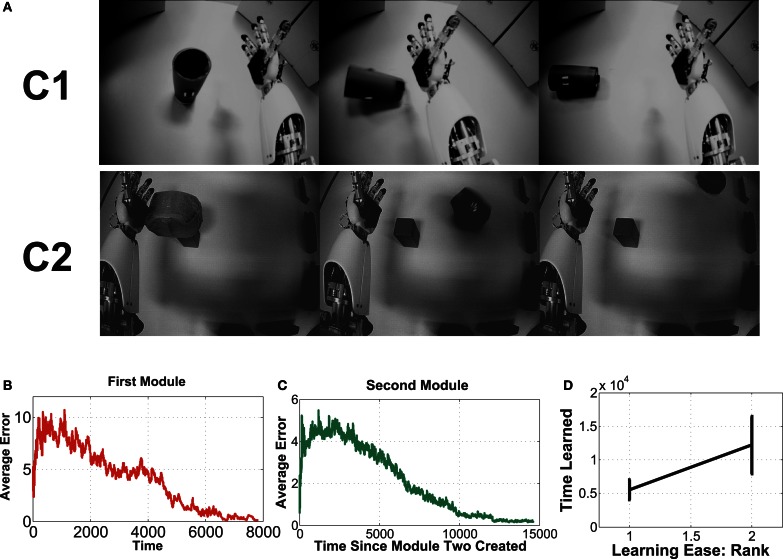
**This experiment uses image sequences from our iCub's cameras, while it moves its arm and interacts with objects.** See text for details.

Three example images from each of the two contexts are shown in Figure 6A. Each episode involves random exploration and an object-robot interaction event, and has between 50 and 250 images. We can say the “topple” context is easier to learn than the other, since the Ω value for the “topple” images is 0.9982, and the Ω value for the “push” images is 0.9988.

For the desired encoding to emerge requires careful setup, since IncSFA (and SFA, generally), applied to images with no preprocessing, is an appearance based vision technique (Turk and Pentland, 1991). To enable learning, we need to keep certain aspects of the images consistent. First, the robot's head is kept stable, so the image background doesn't noticeably shift. If the image shifts, it is possible the features would code for head position. Second, at the beginning of the episode, the object is always placed in the same position.

#### 4.2.1. Setup

The joint angles were quantized into 20 distinct bins, yielding 20 states for each context, leading to 20 different clustering algorithms operating. Each clustering implementation had its maximum number of clusters set to 3. The estimation error threshold, below which the current module is saved and a new module is created, was set to 2.3. The initial ϵ-greedy value was 0.6, with a 0.93 decay multiplier. CCIPCA used learning rate 1/*t* with amnesic parameter 0.4, while the MCA learning rate was 0.01. CCIPCA did variable size dimension reduction by calculating how many eigenvalues would be needed to keep 98% of the input variance — typically this was between 10 and 15—so the 7500 pixels could be effectively reduced to only about 10 dimensions.

Unlike in the synthetic signals experiment, the slowest feature here encodes the context identity, which is to be expected when the input signals from widely different clusters; in a sense this is similar to a multiple rooms case (Mahadevan and Maggioni, 2007), where the features code for room ID. In order to prevent learning progress from continual switching, the following rule was implemented: when the agent decided to remain in its current context, it experienced two subsequent episodes, but when it decided to switch to the other, it only experienced one. In other words, the agent is given more time to learn by staying rather than by switching.

#### 4.2.2. Results

Fifteen experimental runs were performed. Figures 6B–D show results. Part **(B)** shows the average estimation error during the first module's learning, while part **(C)** shows average estimation error for the second. Part **(B)** has a higher error, with more fluctuation than part **(C)**, which mostly involves learning in a single context, since it will learn to quickly disengage away from the already learned context due to boredom punishment. In part **(D)**, one can see the easier representation was indeed mostly learned first (in 14 of the 15 runs, this was the case).

**Figure 7:**
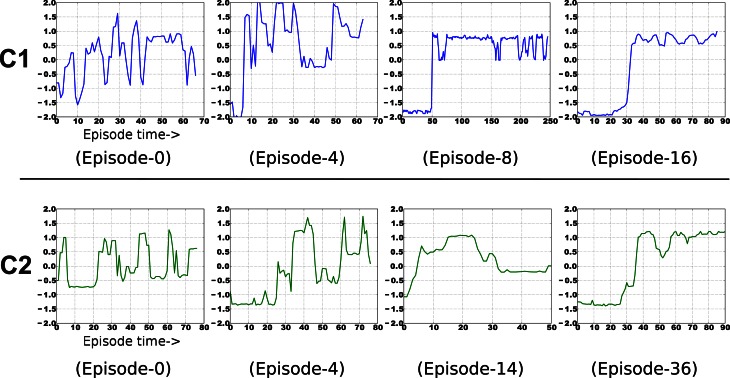
**Example emergence of object-centric slow features for both contexts.** The final result encodes two states of each object—upright or displaced.

Examples of the context-specific representations over time are shown in Figure 7. Both representations eventually encode whether the object is displaced or not. Most of the information in the image sequences can be broken down into three components: a baseline (the background), the object, and the arm. The object changes slower than the arm, so it is preferentially extracted by SFA. Moreover, the object-based features are invariant to the arm's position. Generalization is also possible, in a limited sense. If the arm were replaced by some other object (e.g., a stick), the feature output would not be perturbed. For more robust generalization, a better pre-processing is probably needed, as is typical with appearance-based vision techniques (Cui and Weng, 2000).

Once the features are learned, the feature output space creates a reduced-dimension state space for reinforcement learning techniques, if an external reward is in play. For examples, see our illustrative video of state-space simplification^5^ Kompella et al. (2012b) for an example of using RL to maximize external reward upon the previously learned features.

#### 4.2.3. On concepts

How do the learned representations relate to concepts? CD-MISFA could be the basis for something more substantial in the direction of concept learning, but, by itself, it is limited.

The representations learned by CD-MISFA correspond to compressed descriptions of image feeds, emerging from an eigendecomposition of the covariance of temporally subsequent image differences. In some cases the resulting representations loosely resemble concepts, as when the slowest feature is shown to invariantly capture the state of some object in the images. But we are hesitant to explicitly refer to these representations as concepts, for a number of reasons. First, the notion of concept is itself up for debate. Arguments about what constitutes a concept will necessarily jump disciplinary boundaries, including philosophy, linguistics, and artificial intelligence. We do not wish to wade into this debate however, and we instead concern ourselves with the manner in which an agent or robot, starting with little prior knowledge, might direct its own behavior so as to increase what it knows about the world around it. Second, the types of representations learned by CD-MISFA are generally too low-level to be considered conceptual. For example, if CD-MISFA used intrinsic rewards to guide it to areas which enabled it to develop low-level feature detectors, such as edge detectors (which SFA can learn from a moving fovea Berkes and Wiskott, 2002), would we want refer to the edge detectors or the edges themselves as concepts? Likely not, despite the fact that it could develop from the same learning mechanisms that led to a representation for a toppling event.

### 4.3. Comparison

Baldassarre et al. (2012) recently presented a biologically-constrained model of IM, which is also applicable to developmental robotics. Although the Baldassarre model (TBM) is more closely tied to neuroanatomical function than CD-MISFA, we argue that a number of functional and theoretical drawbacks of TBM make CD-MISFA a superior choice.

TBM was implemented on an iCub robot, and tested in an environment motivated by psychological studies, which includes a box with 3 buttons, 3 doors, and a series of lights. The robot can take 6 oculomotor actions (eyes fixating at either of the 3 buttons or 3 boxes) and 3 arm-motor actions (“reach and press,” “reach and point,” “reach and wave”). The IM reward function is modeled based on illumination change, considered as an automatically extracted salient event, and is a value that decays with recurrence of the salient event. In a learning phase, the model is allowed to explore by selecting any of its oculomotor/arm-motor actions, and observing the result (i.e., the opening of a door). In a test phase, external rewards are “hidden” inside one of the doors, and the goal of the agent becomes: press the correct button to retrieve the reward.

A primary drawback of TBM and its experimental validation is, although it makes use of IM, it is not clear to what extent (if any) IM is necessary for appropriate learning to occur. The model is not tested without an IM reward function, and in principle, the task undertaken would be learnable simply through *random exploration without any IM reward whatsoever*. Conversely, the role of IM in CD-MISFA and its associated experiments is essential, since if CD-MISFA is not presented with intrinsic reward, the model will not stay in any particular context long enough to learn the underlying representations. If CD-MISFA simply explores its environments in a random fashion, it is incapable of learning any meaningful representation.

A major advantage of CD-MISFA over TBM is the former's grounding in the Formal Theory of Fun and Creativity. Whereas the decay of the intrinsic reward value in TBM arbitrarily depends on the number of times the agent repeats a given action, CD-MISFA makes use of the more appropriate learning progress measure. In CD-MISFA, information ceases to be intrinsically rewarding as a function of how and when those visits lose informational value.

Lastly (and perhaps most importantly), TBM does not operate on realistic sensory/motor spaces. Whereas CD-MISFA explicitly shows how IM can operate in a model learning from high-dimensional input streams, and how action selection can operate on low-level motor outputs, TBM only shows how a model of IM can learn a small subset of predefined actions, operating on abstract representations of visual input.

### 4.4. Quantifying the learning cost

We discuss here the measure denoted as Ω, which is used to quantify the learning cost of various types of signals for IncSFA. For simplicity, we consider here signals with similar input-variance but that have a different temporal structure. This assumption allows CCIPCA to approximately have a similar progress for the signals. Therefore, our focus remains here only on the progress of the CIMCA algorithm.

In an approach similar to the proof provided by Peng et al. (2007) for the convergence of MCA, we present here an analysis on quantifying the learning progress of the CIMCA algorithm. For the sake of simplicity, we just consider here only the first output component, but this can trivially be extended for higher output components.

The weight-update rule of CIMCA is given by:

(16) $$\begin{array}{l} w^{mca}(k)=\left(1-\eta^{mca}\right)\;w^{mca}(k-1)\\ \;-\eta^{mca}\;(x(k)\cdot w^{mca}(k-1))\;x(k) \end{array}$$

(17) $$w^{mca}(k)=w^{mca}(k)/\|w^{mca}(k)\|$$

To analyze the “average” dynamics of Equation 16, we reformulate it to a deterministic discrete time (DDT) system by taking the conditional expected value

(18) $$E[w^{mca}(k+1)|w^{mca}(0),x(i),i<k]$$

at each iteration:

(19) $$\begin{array}{l} w^{mca}(k)=\left(1-\eta^{mca}\right)\;w^{mca}(k-1)\\ \;-\eta^{mca}\;E[x(k)x{\left(k\right)}^{T}]w^{mca}(k-1) \end{array}$$

Here, *E*[**xx**^*T*^] is the correlation matrix (*R*) of the input data (**x** ϵ ℛ^*n*^). The weight vector *w*^*mca*^(*k*) is shown to converge to minor component of input data Peng et al. (2007), if the following conditions are satisfied:

(20) $$\begin{array}{l} \;\eta^{mca}\lambda_{1}<0.5,\;\;\;\;||w^{mca}(0)|{|}^{2}=1,\\ 0<\eta^{mca}\leq 0.5,\;\;\;\;w^{mca}{\left(0\right)}^{T}w^{mca}{}^{*}\neq 0 \end{array}$$

where λ_1_ is the largest eigenvalue of *R*, *w*^*mca*^(0) is the initial weight vector and *w*^mca*^ is the eigenvector with the smallest eigenvalue of *R*. Since the correlation matrix *R* is a symmetric non-negative definite matrix, it can be factorized into *QDQ*^−1^, where *Q* is the eigenvector matrix (columns representing unit-eigenvectors *v*_*i*_) and *D* is a diagonal matrix with corresponding eigenvalues (λ_*i*_). In addition, the eigenvectors {*v*_*i*_|*i* = 1, 2, …, *n*} form an orthonormal basis spanning ℛ^*n*^. The weight vector *w*^*mca*^ can then be represented as

(21) $$w^{mca}(k)=\sum_{i=1}^{n}a_{i}(k)v_{i}$$

where *a*_*i*_(*k*) are some constant coefficients.

**Definition 1.** *Given a stationary input distribution* **x** ϵ ℛ^*n*^ *and its eigendecomposition:* {*v*_*i*_, λ_*i*_}, ∀*i* ϵ {1, …, *n*}, *where v denotes the set of eigenvectors and λ their corresponding eigenvalues (such that* λ_1_ > … > λ_*n*_ ≥ 0). *Then, we define* Ω(**x**) *as a measure to indicate the learning progress of CIMCA for the input distribution* **x**.

The following lemmas are useful to derive an analytical expression for Ω. Note that for all the following lemmas to hold true, the convergence conditions in (20) have to be satisfied.

**Lemma 1.** *Let V*_*i*_ *be denoted as*

(22) $$V_{i}=[1-\eta^{mca}-\eta^{mca}\lambda_{i}]$$

*then*,

(23) $$a_{i}(k)=\frac{V_{i}^{k}a_{i}(0)}{\sqrt{\sum_{j}^{n}V_{j}^{2k}a_{j}^{2}(0)}},\forall i\in \{1,2,\ldots ,n\}$$

*Proof*: We prove the result by mathematical induction.

*k* = 1: Substituting (21) in (19) for *k* = 0, we get

$$a_{i}(1)=V_{i}a_{i}(0),\;\;\forall i\in \{1,2,\ldots ,n\}\;$$

At each update, the weight vector *w*^*mca*^(*k*) is normalized according to (17).

(24) $$a_{i}(1)=\frac{V_{i}a_{i}(0)}{\sqrt{\sum_{j}^{n}V_{j}^{2}a_{j}^{2}(0)}},\;\;\forall i\in \{1,2,\ldots ,n\}$$

Therefore, (23) is true for k=1.

*k* = *m*: Assuming the result to be true for some *k* = *m* > 1

$$a_{i}(m)=\frac{V_{i}^{m}a_{i}(0)}{\sqrt{\sum_{j}^{n}V_{j}^{2m}a_{j}^{2}(0)}},\;\;\forall i\in \{1,2,\ldots ,n\}$$

let *P* denote

$$P=\sqrt{\sum_{j}^{n}V_{j}^{2m}a_{j}^{2}(0)}\;$$

*k* = *m* + 1: Substituting (21) in (19) for *k* = *m*, we get

(25) $$a_{i}(m+1)=V_{i}a_{i}(m)=\frac{V_{i}^{m+1}a_{i}(0)}{P}$$

Upon normalizing,

$$\begin{array}{l} a_{i}(m+1)=\frac{\frac{V_{i}^{m+1}a_{i}(0)}{P}}{\sqrt{\sum_{j}^{n}\frac{V_{j}^{2m+2}a_{j}^{2}(0)}{P^{2}}}}\\ \;=\frac{V_{i}^{m+1}a_{i}(0)}{\sqrt{\sum_{j}^{n}V_{j}^{2m+2}a_{j}^{2}(0)}},\;\;\;\forall i\in \{1,2,\ldots ,n\} \end{array}$$

which is same as substituting *k* = *m* + 1 in (23). Therefore, by the principle of mathematical induction the result (23) is true for any *k* > 1.            □

**Lemma 2.** *Let* σ_*i*_ *be denoted as*

(26) $$\sigma_{i}=[1-\frac{\eta^{mca}(\lambda_{i}-\lambda_{n})}{1-\eta^{mca}-\eta^{mca}\lambda_{n}}]$$

*then*,

(27) $$0<\sigma_{1}<\cdots <\sigma_{n-1}<1$$

*Proof:* If we show that

(28) $$0<\frac{\eta^{mca}(\lambda_{i}-\lambda_{n})}{1-\eta^{mca}-\eta^{mca}\lambda_{n}}<1$$

then the condition (27) is straightforward.

We first prove the left inequality. Clearly, since λ_1_ > … > λ_*n*_ ≥ 0 and 0 < η^*mca*^ ≤ 0.5, the numerator

(29) $$\eta^{mca}(\lambda_{i}-\lambda_{n})>0,\;\forall i\in \{1,\ldots ,n-1\}$$

and the denominator

(30) $$\begin{array}{l} 1-\eta^{mca}-\eta^{mca}\lambda_{n}>1-\eta^{mca}-\eta^{mca}\lambda_{1}\\ \;>0.5-\eta^{mca}\lambda_{1},\;\;∵\eta^{mca}<0.5\\ \;>0,\;\;\;\;\;\;\;∵\eta^{mca}\lambda_{1}<0.5 \end{array}$$

To prove the right inequality, it holds

$$\begin{array}{c} \begin{array}{l} \text{iff,}\;\;\;\;\;\;\eta^{mca}(\lambda_{i}-\lambda_{n})\;\;<\;\;1-\eta^{mca}-\eta^{mca}\lambda_{n}\\ \text{iff,}\;\;\;\;\;\;\eta^{mca}(\lambda_{1}-\lambda_{n})\;\;<\;\;1-\eta^{mca}-\eta^{mca}\lambda_{n}\\ \text{iff,}\;\;\;\;\;\;\;\;\;\;\;\;\;\;\;\;\eta^{mca}\lambda_{1}\;\;<\;\;1-\eta^{mca}\\ \text{iff,}\;\;\;\;\;\;\;\;\;\;\;\;\;\;\;\;\;\;\;\;\;\;0.5\;\;<\;\;1-\eta^{mca},\;\;\;\text{which is true} \end{array}\\ \;□ \end{array}$$

**Lemma 3.** *Let* $C_{i}=\left[\frac{a_{i}(0)}{a_{n}(0)}\right]$ *then*,

(31) $$a_{i}(k)=C_{i}\sigma_{i}^{k}a_{n}(k),\;\;\forall i\in \{1,\ldots ,n-1\}$$

(32) $$a_{n}(k)=\frac{1}{\sqrt{\sum_{j}^{n-1}\sigma_{j}^{2k}C_{j}^{2}+1}}$$

*Proof*: Using Equation (23) and the condition (30), we get

$$\begin{array}{l} \;\frac{a_{i}(k+1)}{a_{n}(k+1)}=\left[\frac{1-\eta^{mca}-\eta^{mca}\lambda_{i}}{1-\eta^{mca}-\eta^{mca}\lambda_{n}}\right]\cdot \left[\frac{a_{i}(k)}{a_{n}(k)}\right],\\ \forall i\in \{1,\ldots ,n-1\}=\left[1-\frac{\eta^{mca}(\lambda_{i}-\lambda_{n})}{1-\eta^{mca}-\eta^{mca}\lambda_{n}}\right]\cdot \left[\frac{a_{i}(k)}{a_{n}(k)}\right]\\ \;=\sigma_{i}\cdot \left[\frac{a_{i}(k)}{a_{n}(k)}\right]\\ \;=\sigma_{i}^{k+1}\cdot \left[\frac{a_{i}(0)}{a_{n}(0)}\right] \end{array}$$

This implies,

$$a_{i}(k)=C_{i}\sigma_{i}^{k}a_{n}(k),\;\;\forall i\in \{1,\ldots ,n-1\}\;$$

Using the result from Lemma 1 and substituting for i = n, we get

$$\begin{array}{c} \begin{array}{l} a_{n}(k)=\frac{V_{n}^{k}a_{n}(0)}{\sqrt{\sum_{j}^{n}V_{j}^{2k}a_{j}^{2}(0)}}\\ \;=\frac{1}{\sqrt{{\sum_{j}^{n-1}\left(\frac{V_{j}}{V_{n}}\right)}^{2k}{\left(\frac{a_{j}(0)}{a_{n}(0)}\right)}^{2}+1}}\\ \;=\frac{1}{\sqrt{\sum_{j}^{n-1}\sigma_{j}^{2k}C_{j}^{2}+1}} \end{array}\\ \;□ \end{array}$$

Lemma 3 gives an expression for each of the coefficients. Since *a*_*n*_(*k*) is bounded (0 < *a*_*n*_(*k*) < 1), *a*_*i*_(*k*)'s (∀*i* ϵ {1, …, *n* − 1}) belong to a family of exponential-decay functions: *C*_*i*_*a*_*n*_(*k*)*e*^−*kln*(1/σ_*i*_)^. Therefore,

(33) $$\underset{k\to \infty }{\lim}a_{i}(k)=0,\;\;\forall i\in 1,\ldots ,n-1$$

(34) $$\underset{k\to \infty }{\lim}a_{n}(k)=1$$

Therefore, from (21) *w*^*mca*^(*k*) converges to the minor-component vector *v*_*n*_.

**Theorem 1.** *Let* τ^1/2^_*i*_ *denote the half-life period of a*_*i*_(*k*), *then the following inequality holds:*

(35) $$\tau_{1}^{1/2}<\cdots <\tau_{n-1}^{1/2}$$

*Proof:* Since *a*_*n*_(*k*) is bounded (0 < *a*_*n*(*k*) < 1), *a*_*i*_(*k*)'s (∀*i*_ ϵ {1, …, *n* − 1}) belong to a family of exponential-decay functions: *C*_*i*_*a*_*n*_(*k*)*e*^−*kln*(1/σ_*i*_)^. Half-life period τ^1/2^_*i*_ is the time when the value *a*_*i*_(*k*) becomes equal to half its initial value. Therefore,

$$C_{i}a_{n}(k)\sigma_{i}^{k}=C_{i}a_{n}(0)/2$$

Using Lemma 3 and simplifying we get,

(36) $$k=-\frac{ln(2)}{ln\sigma_{i}}+\frac{0.5}{ln\sigma_{i}}\times \frac{\sum_{j}^{n-1}\sigma_{j}^{2k}C_{j}^{2}+1}{\sum_{j}^{n-1}C_{j}^{2}+1}$$

Let us denote the term $\frac{\sum_{j}^{n-1}\sigma_{j}^{2k}C_{j}^{2}+1}{\sum_{j}^{n-1}C_{j}^{2}+1}$ as ξ. It is clearly evident by using Lemma 2 that for *k* > 0, 0 < ξ < 1 and ξ is a monotonically decreasing function w.r.t *k*. However, for larger values of *k* and for consecutive σ_*i*_'s, ξ can be assumed to be a constant. Substituting the term ξ in Equation (36), we get

(37) $$\begin{array}{l} \tau_{i}^{1/2}=-\frac{ln(2)-0.5*ln(\xi )}{ln\sigma_{i}}\\ \;=\frac{ln(2)-0.5*ln(\xi )}{ln(1/\sigma_{i})} \end{array}$$

Therefore, from Equation (37) and Lemma 2 we have,

(38) $$\begin{array}{c} \tau_{j-1}^{1/2}<\tau_{j}^{1/2},\;\forall j\in \{2,\ldots ,n-1\}\\ \;□ \end{array}$$

Theorem 1 gives the order in which the individual components *a*_*i*_(*k*) decay over time.

**Theorem 2.** *Given two input distributions* **x**^1^, **x**^2^ ϵ ℛ^*n*^ *and the eigendecomposition of their corresponding expected correlation-matrix:* {*v*^1^_*i*_, λ^1^_*i*_}, {*v*^2^_*i*_, λ^2^_*i*_}, ∀*i* ϵ {1, …, *n*}, where *v* denotes the set of eigenvectors and λ their corresponding eigen-values (λ_1_ > … > λ_*n*_ ≥ 0). *For an* η^*mca*^ *that satisfies*,

(39) $$\eta^{mca}\lambda_{1}^{1}<0.5\;,\;\;\eta^{mca}\lambda_{1}^{2}<0.5$$

*Then, the signal with a lower* σ_*n* − 1_ *will have quicker convergence and therefore quicker learning progress*.

*Proof:* From Theorem 1, it is clear that, the weight-vector *w*^*mca*^(*k*) converges to the minor component *v*_*n*_ when the penultimate coefficient *a*_*n* − 1_(*k*) tends to 0. Therefore, a signal with lower σ_*n* − 1_ will have a lower half-life period τ^1/2^_*n* − 1_ and hence the weight-vector *w*^*mca*^(*k*) converges quicker.        □

**Definition 2.** *We therefore define* Ω(**x**) *as a measure to indicate the learning progress of CIMCA for an input-distribution* **x** *equal to* σ^*th*^_*n* − 1_ *value, that is*,

(40) $$\Omega (x)=\left[1-\frac{\eta^{mca}(\lambda_{n-1}-\lambda_{n})}{1-\eta^{mca}-\eta^{mca}\lambda_{n}}\right]$$

## 5. Conclusions

A CD-MISFA agent autonomously explores multi-context environments. Compact context representations are learned from high-dimensional inputs through incremental slow feature analysis. Intrinsic rewards for measurable learning progress tell the agent which context is temporarily “interesting,” and when to actively engage in/disengage from a context or task. Such mechanisms are necessary from a computational perspective, and biological systems have evolved methods of achieving similar functional roles. In particular, while cortical regions of the brain are involved in unsupervised learning from sensory data (among other things), neuromodulatory systems are responsible for providing intrinsic rewards through dopamine, and regulating levels of attention to allow for task engagement and disengagement through norepinephrine. As artificial and robotic agents become increasingly sophisticated, they will not only look to biological solutions for inspiration, but may begin to resemble those solutions simply through the pressure of computational constraints.

### Conflict of interest statement

The authors declare that the research was conducted in the absence of any commercial or financial relationships that could be construed as a potential conflict of interest.

## Footnotes

^1^A detailed comparison with the model of Baldassarre *et al* is presented in Section 4.3.

^2^We note that there are some similarities with the *options* framework Sutton et al. (1999) here. One could link the start condition idea to an initiation state, and the end condition would correspond to having a termination probability of one at some state, and zero elsewhere. Indeed, one can view the problem of representation learning as analogous to abstraction learning in options, which remains an important open problem. However, we do not need to formalize contexts as options in this paper.

^3^To our knowledge, this has not been tested yet.

^4^We use “time step” in a local sense—it refers to one of 1000 steps that make up each context. We will use “time” to refer to global time (over all contexts).

^5^http://www.idsia.ch/~luciw/videos/IncSFAArm.mp4
